# Dance/Movement Group Therapy for Older Adults With Dementia

**DOI:** 10.1093/geroni/igaf122.848

**Published:** 2025-12-31

**Authors:** YinHui Hong, Hao-Min Chen

**Affiliations:** University of Taipei, Taipei, Taipei, Taiwan (Republic of China); Texas A&M University, Central Texas, Killeen, Texas, United States

## Abstract

Dementia significantly affects cognitive, psychosocial, and neuroendocrine functioning in older adults. Globally, 8% of individuals over 60 and 7.99% of those over 65 in Taiwan are diagnosed with dementia. This study examined the therapeutic process of older adults with dementia participating in a Dance/Movement Therapy (DMT) group at a Taiwanese hospital. The intervention aimed to enhance bodily awareness, emotional expression, and social connection through semi-structured movement activities. Participants attended 20 sessions, each lasting 1.5 hours, held twice weekly. A social constructionist and narrative inquiry approach was adopted. Using purposeful sampling, nine older adults with dementia of various stages (mean age = 71.8) and their caregivers were recruited. Data sources included video and audio recordings, progress notes, and field observations. Narrative analysis identified three key themes: (1) a shift from unconscious bodily presence to heightened awareness and support; (2) progression from rigid postures to fluid movement, and (3) transformation from isolation to social resonance and connection. Findings underscore the importance of leveraging participants’ strengths, resilience, and inherent capacities, helping them rediscover abilities they may not have previously recognized. This empowerment fostered engagement, agency, and enhanced well-being. The key takeaway from this study is that DMT offers a promising non-pharmacological approach for individuals with dementia, emphasizing the importance of preserving functionality, fostering individual experiences, and enhancing relational connections. It encourages a paradigm shift in focus from diagnosis and decline to holistic care centered on people and their interactions, underscoring the transformative potential of the therapeutic process.

